# Short-and long-term outcomes of one-stage versus two-stage gastrectomy for perforated gastric cancer: a multicenter retrospective propensity score-matched study

**DOI:** 10.1186/s12957-023-03283-4

**Published:** 2024-01-03

**Authors:** Junling Zhang, Kexuan Li, Zongnai Zhang, Guochao Zhang, Shupeng Zhang, Yinming Zhao, Zhaoya Gao, Haiyun Ma, Yong Xie, Jinsheng Han, Li Zhang, Baoliang Zhang, Yang Liu, Tao Wu, Yingchao Wu, Yi Xiao, Xin Wang

**Affiliations:** 1 Department of Gastrointestinal Surgery, Peking University First Hospital, No.8, Xishiku Street, Xicheng District, Beijing, 100034 P. R. China; 2 Division of Colorectal Surgery, Department of General Surgery, Peking Union Medical College Hospital, No.1 Shuaifuyuan Wangfujing Street, Dongcheng District, Beijing, 100730 P. R. China; 3 Department of General Surgery, Civil Aviation General Hospital, No.1A, Gaojing, Chaoyangmenwai Avenue, Chaoyang District, Beijing, 100123 P. R. China; 4 Department of General Surgery, China-Japan Friendship Hospital, East Yinghuayuan Street, Chaoyang District, Beijing, 100029 P. R. China; 5 Department of General Surgery, Tianjin Fifth Central Hospital, No.41 Zhejiang Road, Binhai New Area, Tianjin, 300450 P. R. China; 6 Department of General Surgery, Beijing Jingmei Group General Hospital, No.18, Heishan Street, Mentougou District, Beijing, 102399 P. R. China; 7 Department of General Surgery, Peking University Shougang Hospital, No.9, Jinyuanzhuang Road, Shijingshan District, Beijing, 100144 P. R. China; 8 Department of General Surgery, Beijing Miyun District Hospital, Miyun District, No.36 Mixi Road, Beijing, 101500 P. R. China; 9 Department of General Surgery, Hebei General Hospital, No.348 Heping West Road, Xinhua District, Shijiazhuang, Hebei Province 050051 P. R. China; 10 Department of General Surgery, Cangzhou Integrated Traditional Chinese and Western Medicine Hospital, No.31 Huanghe West Road, Yunhe District, Cangzhou, Hebei Province 061011 P. R. China; 11 Department of General Surgery, First Hospital of Qinhuangdao, No. 258 Wenhua Road, Haigang District, Qinhuangdao, Hebei Province 066000 P. R. China; 12 Department of General Surgery, Tangshan Workers’ Hospital, No.27, Wenhua Road, Tangshan, Hebei Province 063003 P. R. China; 13 Department of General Surgery, North China University of Science and Technology Affiliated Hospital, No.73 Jianshe South Road, Lubei District, Tangshan, Hebei Province 063000 P. R. China

**Keywords:** Perforated gastric cancer (PGC), Radical gastrectomies, Propensity score matching (PSM), Overall survival (OS), Lymphadenectomy

## Abstract

**Objective:**

There is no scientific consensus about the treatment of perforated gastric cancer (PGC). Therefore, the aim of this study was to investigate which is the better treatment option for PGC between the single-stage and two-stage strategies.

**Methods:**

All 81 PGC patients from 13 medical institutions were retrospectively enrolled in this study. The PGC patients who underwent R0 gastrectomy were divided into one-stage surgery and two-stage surgery groups. The clinicopathological characteristics of the two groups were compared, and 415 regular gastric cancer patients without perforation were randomly selected as a control. The propensity score matching (PSM) method was used to find matched regular GC patients with similar clinicopathological parameters. The OS (overall survival) and the number harvested lymph nodes from PGC patients and regular GC patients were compared.

**Results:**

Compared with PGC patients who underwent one-stage surgery, those who underwent two-stage surgery harvested significantly more lymph nodes [31(27, 38) vs 17 (12, 24), *P* < 0.001], required less blood transfusion [0 (0, 100) vs 200 (0, 800), *P* = 0.034], had a shorter ICU stay [0 (0, 1.5) vs 3 (0, 3), *P* = 0.009], and had a significantly better OS (Median OS: 45 months vs 11 months, *P* = 0.007). Compared with propensity score-matched regular GC patients without perforation, PGC patients who underwent one-stage gastrectomy had a poorer quality of lymphadenectomy [17 (12, 24) vs 29 (21, 37), *P* < 0.001] and suffered a worse OS (Median OS: 18 months vs 30 months, *P* = 0.024). Conversely, two-stage gastrectomy can achieve a comparable quality of lymphadenectomy (*P* = 0.506) and a similar OS (*P* = 0.096) compared to propensity score-matched regular GC patients.

**Conclusions:**

For PGC patients in poor condition, two-stage treatment is a better option when D2 radical gastrectomy cannot be achieved in emergency surgery, based on our findings that two-stage gastrectomy could provide PGC patients with a better quality of lymphadenectomy and a better OS.

## Instruction

Gastrointestinal perforation caused by gastric cancer is a rare complication with significant in-hospital mortality. Less than 3% of gastric cancer patients develop perforation, which is the second most common oncologic emergency after severe bleeding [1, 2]. The spontaneous perforation brought on by gastric cancer might result in serious abdominal infections that can even be fatal [3]. When diffuse peritonitis due to gastrointestinal perforation is diagnosed, emergency surgery must be scheduled as soon as possible. As a result, a detailed preoperative examination is often omitted [4].

Any gastric perforation should raise the suspicion of gastric cancer. However, only one third of patients with perforated gastric cancer (PGC) can be diagnosed preoperatively. The pathological diagnosis of malignancy can only be determined by intraoperative pathological biopsy or postoperative pathology. As a result, in emergency surgery, it would be a challenge for surgeons to achieve radical gastrectomy with high quality of lymphadenectomy, either because the pathological diagnosis of malignancy is not confirmed or because the patient's poor condition does not allow for extended surgery.

Unfortunately, there is currently no widely accepted standardized treatment for PGC patients, and surgeons are challenged to make surgical strategies for them. In general, there are two strategies for the treatment of perforated gastric cancer: one is to perform radical gastrectomy directly based on intraoperative findings or frozen pathology results, defined as the one-stage strategy; the other is to perform primary perforation repair followed by second-stage radical gastrectomy, defined as the two-stage strategy. However, it is currently controversial as to which procedure provides a better outcome for PGC patients. The two-stage procedure may be a better option for these patients. First, the pathological diagnosis and tumor staging can be determined during the initial perforation repair. Subsequently, a radical gastrectomy can be planned when the patient has recovered from the peritonitis.

In this retrospective study, we aim to investigate the impact of different surgical strategies on the short and long term prognosis of PGC patients, and which type of surgical strategy may provide a better outcome and what the mechanisms may be.

## Materials and methods

### Study population and inclusion/exclusion criteria

We retrospectively collected the records of 81 patients with PGC from thirteen high-level medical institutions between January 2010 and June 2021. All patients' biopsies were confirmed as gastric adenocarcinoma. Patients were excluded if (1) gastric perforation was related to endoscopic submucosal dissection (ESD); (2) clinical parameters, clinical follow-up or survival information were missing; (3) postoperative pathology confirmed a pathological gastric perforation caused by pathological types other than gastric adenocarcinoma, such as gastric lymphoma or metastatic tumor of other origins; (4) GC accompanied by perforation of a concomitant gastric ulcer. In the end, 74 patients were enrolled in the analysis.

At the same time, a total of 2273 regular GC patients from Peking University First Hospital (PKUFH) and Peking Union Medical College Hospital (PUMCH) were studied, gastric adenocarcinoma was confirmed in all patients' biopsies, and all patients were sorted by order of hospital admission. We generated 500 random numbers as a control subgroup of the cohort using the RAND function in Microsoft Excel. The randomly selected 500 cases excluded early gastric cancer patients underwent ESD, advanced gastric cancer patients treated with chemotherapy only, patients with missing clinical data, clinical follow-up and survival information. Finally, 415 GC patients without perforation were randomly selected from the clinical database as a control subgroup.

The clinical parameters of the patients we collected included the gender and the age of the patients, surgical procedure, tumor diameter, tumor differentiation, tumor location (upper middle or lower 1/3 of the stomach), the depth of invasion (T stage), lymph node metastasis (N stage), distant metastasis (M stage), curability, node dissection (D-number), blood transfusion volume (for two-stage gastrectomy, transfusion volume was composed of the total transfusion volume of both the emergency and the radical operations, including red blood cells and plasma), harvested lymph nodes, hospital mortality, chemotherapy status, and postoperative survival.

### Surgery and outcome measures

All 13 participating medical institutions are certified as high-level comprehensive tertiary care hospitals by the Chinese National Health Authorities. Surgical resections were divided into subtotal and total gastrectomy, and D1, D1 + or D2 lymphadenectomy was performed at each center according to the lymph node classification of the Japanese Gastric Cancer Association [5]. For total gastrectomy, the D1 lymphadenectomy includes: Nos. 1–7. The D1 + lymphadenectomy includes: D1 + Nos. 8a, 9, 11p. The D2 lymphadenectomy includes: D1 + Nos. 8a, 9, 11p, 11d, 12a. For distal gastrectomy, the D1 lymphadenectomy includes: Nos. 1, 3, 4sb, 4d, 5, 6, 7. The D1 + lymphadenectomy includes: D1 + Nos. 8a, 9. The D2 lymphadenectomy includes: D1 + Nos. 8a, 9, 11p, 12a [5]. In addition, completeness of gastrectomy was determined using the UICC residual tumor (R) classification, where R0 represents no residual cancer cells, R1 represents microscopic residual cancer cells, and R2 represents macroscopic residual cancer tissue, respectively [6]. Overall survival (OS) was assessed by telephone interview.

### Propensity score-matching (PSM) analysis

A PSM analysis was employed to control for potential baseline bias in the baseline of PGC patients or regular GC patients who underwent R0 surgery. This approach removed the bias associated with a multivariate model and analysis [7]. The covariates included age, gender, tumor diameter, tumor differentiation, T staging, N staging, M staging, anatomical location of the tumor, and carcinoembryonic antigen (CEA) level, and the matching tolerance was set to 0.2. First, individual propensity scores were calculated using logistic regression analysis SPSS 26.0 (IBM Corp., NY, USA). Then, based on the propensity scores, optimal 1:1 matching was performed between the PGC and regular GC groups. After matching, the distribution of variables was similar in both groups.

### Data analysis

The clinical, surgical, and pathological records of all patients with gastric cancer perforation were reviewed. Patients were retrospectively evaluated for the following factors: gender; age; anatomical location of the tumor in the stomach (defined as upper, middle, or lower third); depth of invasion (T3 or T4); size of the resected specimen; tumor differentiation; TNM staging; neoadjuvant chemotherapy and adjuvant chemotherapy. Quantitative parameters in the study were presented as mean ± standard deviation. When data were not normally distributed, median and interquartile ranges were used instead. Student's t-test was used to compare quantitative data between two groups; and the Mann–Whitney U test was used for skewed distributions by using SPSS 26.0 (IBM Corp., NY, USA). The ggplot2 package in R 4.0 software (https://www.r-project.org/) was used to plot OS curves. The Kaplan–Meier method (the log-rank test) was used to compare the OS times between two groups by using SPSS 26.0 (IBM Corp., NY, USA). Differences were considered significant at *P* < 0.05.

## Results

### Clinicopathological parameters of patients

**Fig. 1:**
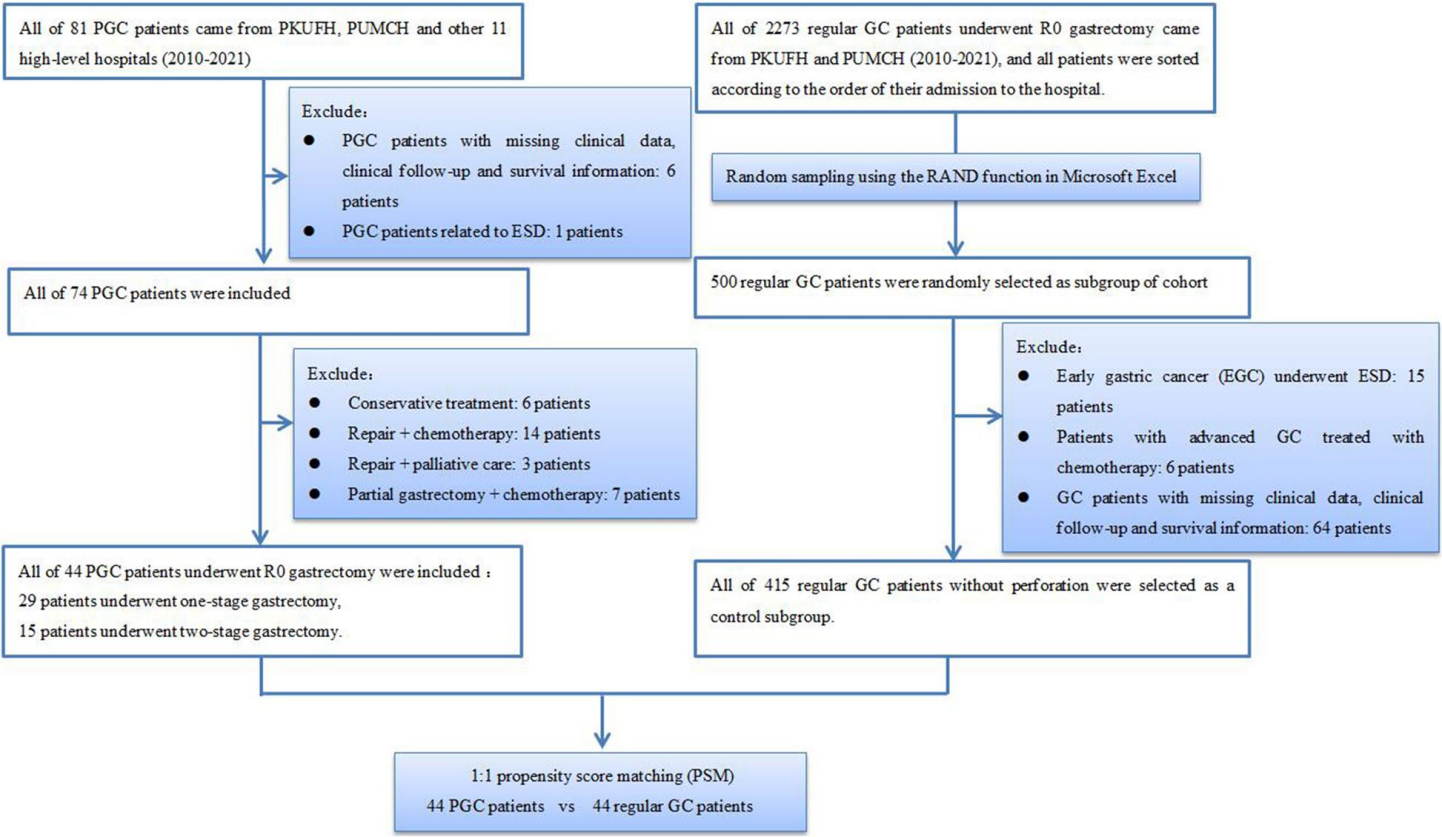
The schematic diagram of this study

**Fig. 2:**
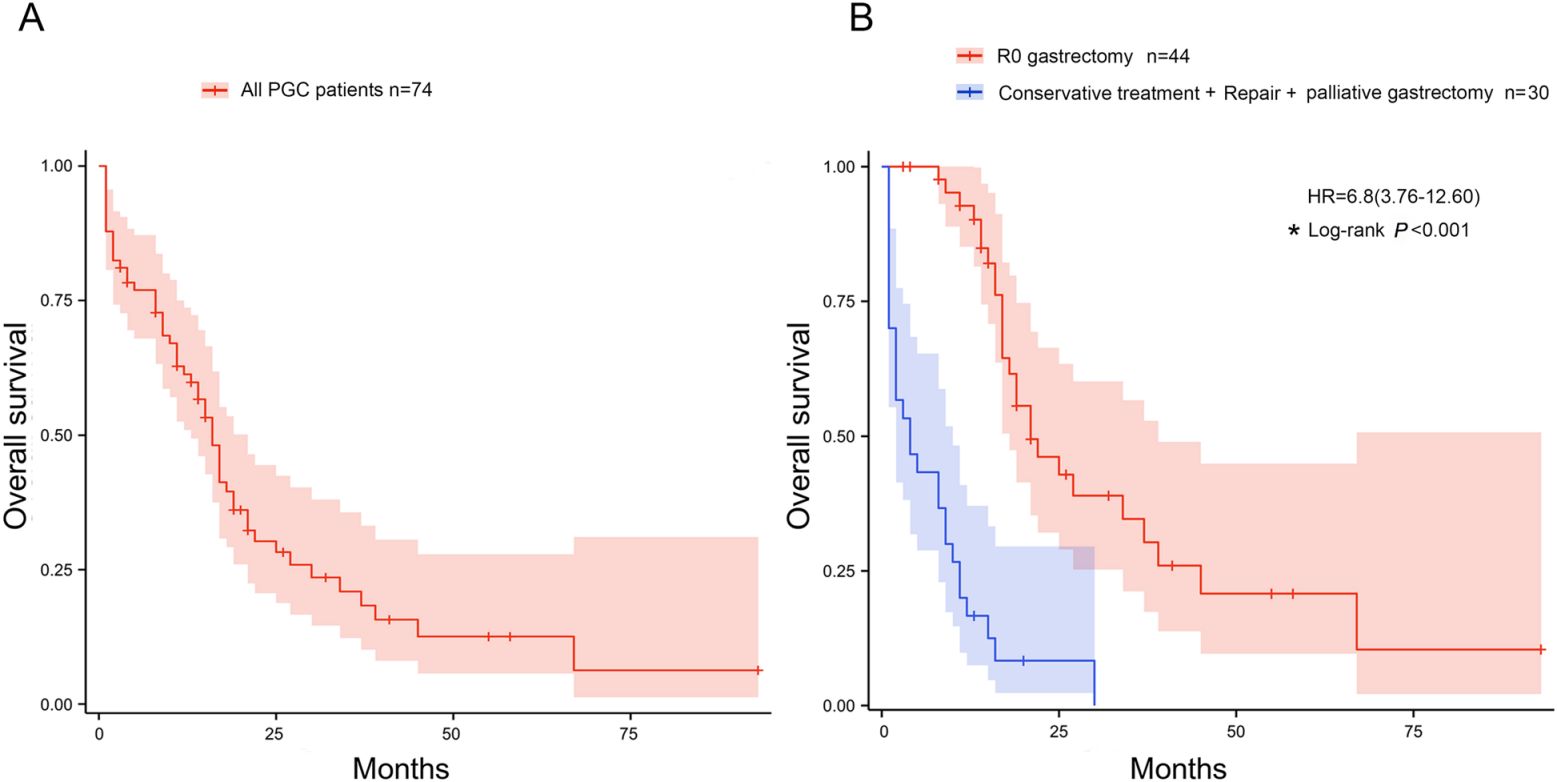
Overall survival of PGC patients who underwent R0 gastrectomy was much better than patients who underwent conservative treatment, repair or palliative partial gastrectomy. **A** The Kaplan–Meier curve of all 74 PGC patients, and the median survival for the whole cohort was 16 months (95% CI: 13–21 months). **B** Compared with patients who underwent conservative treatment, repair or palliative partial gastrectomy gastrectomy (median survival time was 4 months, 95% CI: 2–10 months), patients who underwent R0 gastrectomy had a significantly better OS (median survival time was 21 months, 95% CI: 18–39 months, *P* < 0.001)

A total of 74 patients with perforated gastric cancer treated between 2010 and 2021 were included in the study. Clinicopathological data were obtained from the study group, which consisted of 53 males and 21 females with a mean age of 66.07±12.39 years (range 39 to 92 years) (Table 1). The median follow-up was 14 months (range 1 to 93 months). Of these 74 patients, only seven patients had pathological findings prior to perforation and one patient had received chemotherapy prior to perforation (Fig. 1). The median OS for the whole cohort was 16 months (95% CI: 13-21 months, Fig. 2A).

**Table 1:** Baseline characteristics of all PGC patients

Clinical parameters	Number of Patients
**Age**	
Range (yr)/Mean ± SD	39–92/66.07 ± 12.39
**Gender**	
Male	71.62% (53/74)
Female	28.38% (21/74)
**Preoperative pathological diagnosis**	
Yes	13.51% (10/74)
No	86.49% (64/74)
**Chemotherapy before onset of perforation**	
Yes	1.35% (1/74)
No	98.65% (73/74)
**Location of cancer**	
Upper and middle 1/3	16.22% (12/74)
Lower 1/3	83.78% (62/74)
**Lymph node metastasis**	
N0	12.16% (9/74)
N1-3	87.84% (65/74)
**T stage**	
T3	1.35% (1/74)
T4a	94.59% (70/74)
T4b	4.05% (3/74)
**TNM stage**	
II-B (T3N1M0, T4a N0 M0)	13.51% (10/74)
III-A/B/C(T4a-b N1-3 M0)	45.95% (34/74)
IV (Tany Nany M1)	40.54% (30/74)
**Treatments**	
Conservative treatment	8.11% (6/74)
Repair + chemotherapy	18.92% (14/74)
Repair + Palliative care	4.05% (3/74)
Palliative partial gastrectomy + chemotherapy	9.46% (7/74)
One-stage radical gastrectomy	5.41% (4/74) (Total gastrectomy)
	33.78% (25/74) (Subtotal gastrectomy)
Two-stage radical gastrectomy	20.27% (15/74) (Subtotal gastrectomy)

Of all patients, 10 (13.51%, 10/74) patients were in stage IIB, 34 (45.95%, 34/74) patients in stage IIIA-B and 30 (40.54%, 30/74) patients in stage IV. Due to limited peritonitis and advanced stage, six (8.11%, 6/74) patients chose conservative treatment as their first option. Another 17 (22.97%, 17/74) patients with IV stage GC were treated by perforation closure and omental patch repair; one (1.35%, 1/74) of whom died within one week after surgery. After recovery from peritonitis, 14 (18.92%, 14/74) cases of them received maintenance chemotherapy and nutritional support, one (1.35%, 1/74) case with liver metastases treated by transcatheter arterial chemoembolization, and another (1.35%, 1/74) case was diagnosed with Krukenberg tumor, and she underwent a bilateral ovariectomy. Seven (9.46%, 7/74) patients with metastatic gastric cancer were treated with palliative partial gastrectomy and maintenance chemotherapy (Table 1). Radical gastrectomy was performed in 44 (59.46%, 44/74) patients with stage II-III gastric cancer. In these patients, there was no imaging evidence of distant metastases or visible peritoneal metastases. Of the 44 cases, 29 (65.91%, 29/44) cases were completed in one stage (categorized as the one-stage gastrectomy group). Other 15 (34.09%, 15/44) cases underwent an omental patch repair surgery and another following radical gastrectomy with regional lymphadenectomy (categorized as the two-stage gastrectomy group, Table 1). In the two-stage cohort, six (40.00%, 6/15) patients received 2-4 cycles of perioperative chemotherapy prior to radical gastrectomy, and two (13.33%, 2/15) of these patients received intraoperative sustained-release fluorouracil implants after radical resection.

### Overall survival of PGC patients (R0 gastrectomy vs. repair or palliative gastrectomy or conservative treatment)

In this study, 44 PGC patients underwent R0 gastrectomy. The other 24 patients with stage IV PGC underwent omental patch repair or palliative gastrectomy followed by maintenance chemotherapy. At the same time, another 6 patients just chose conservative treatment. The log-rank test showed that PGC patients who achieved R0 gastrectomy (median OS time is 21 months) had a better OS than patients who underwent repair, palliative gastrectomy or conservative treatment (median OS time is 4 months, Fig. 2B, *P*<0.001).

### Comparison between one-stage and two-stage gastrectomy

**Fig. 3:**
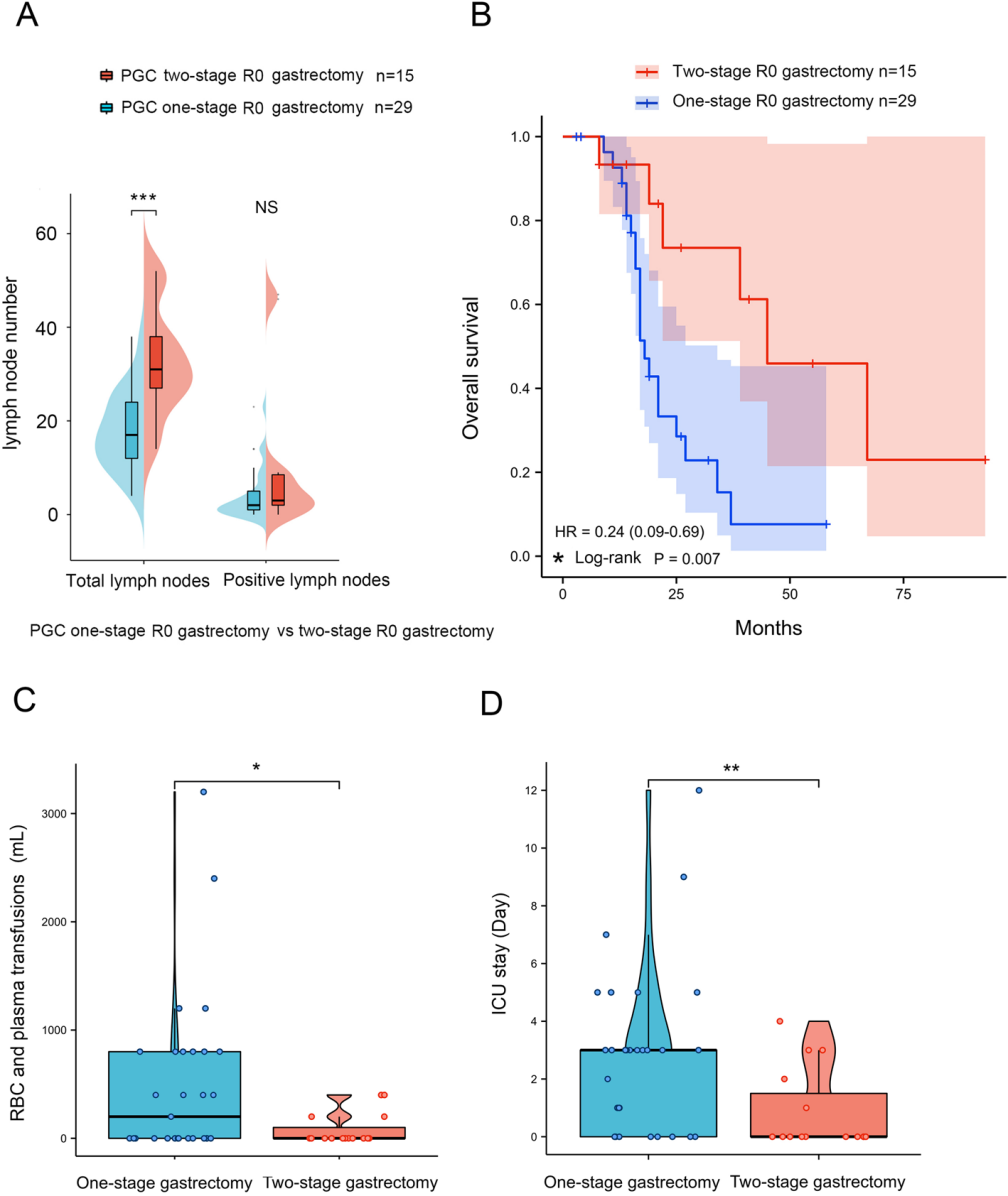
Two-stage gastrectomy harvested more lymph nodes, and patients in the two-stage gastrectomy group had better short-and long-term outcomes. **A** Compared with one-stage gastrectomy, two-stage gastrectomy harvested more lymph nodes (*P* < 0.001). **B** Compared with patients in the one-stage group, PGC patients who underwent two-stage R0 gastrectomy had a much better OS (*P* < 0.007). **C** Compared with patients in the one-stage group, patients who underwent two-stage gastrectomy had a substantially lower volume of RBC and plasma transfusions (*P* = 0.034). **D** In addition, patients who underwent two-stage gastrectomy had a significantly shorter ICU stay (*P* = 0.009)

In this study, 44 patients achieved R0 gastrectomy. The median follow-up was 17.5 months (range from 3 to 93 months). Among them, 29 patients underwent one-stage gastrectomy, and 18 patients achieved D2 gastrectomy (62.07%, 18/29), while other 11 patients (37.93%, 11/29) achieved only D1 or D1+ gastrectomy. In the two-stage group, all 15 patients achieved D2 gastrectomy (100.00%, 15/15). The median time between primary surgery and radical gastrectomy was 8 weeks (ranging from 3 weeks to 18 weeks). The result showed that two-stage gastrectomy could achieve a higher rate of D2 gastrectomy (Table 2, *P*=0.008). In the two-stage group, 31(27, 38) lymph nodes were harvested from the radical gastrectomy. However, only 17(12, 24) lymph nodes were harvested in the one-stage group, (Table 2, Fig. 3A, *P*<0.001). However, the number of positive lymph nodes was similar in both groups (Table 2, Fig. 3A, *P*=0.158).

**Table 2:** Baseline characteristics of PGC patients underwent one-stage vs two-stage R0 surgery

Clinical parameters	One-stage gastrectomy (*n* = 29 patients)	Two-stage Gastrectomy (*n* = 15 patients)	Statistical value	*P* value
Age	63.72 ± 11.99	62.67 ± 12.16	T = 0.276	0.784
Gender (male)	75.86% (22/29)	80.00% (12/15)	χ^2^ = 0.096	0.756
Tumor diameter (≥ 5 cm)	62.07% (18/29)	93.33% (14/15)	χ^2^ = 4.872	0.027
Daily use of antiplatelet drugs	27.59% (8/29)	20.00% (3/15)	χ^2^ = 0.303	0.582
Tumor differentiation (SRCC or Poorly/all)	13.80% (4/29)	6.67% (1/15)	χ^2^ = 0.499	0.480
T staging (T4)	100.00% (29/29)	93.33% (14/15)	–-	0.341^a^
N staging (N positive)	20.69% (6/29)	13.33% (2/15)	χ^2^ = 0.360	0.549
M staging (M positive)	0.00% (0/29)	0.00% (0/15)	–-	0.999^a^
Anatomical location ( Lower 1/3)	79.31% (23/29)	86.67% (13/15)	χ^2^ = 0.360	0.549
HGB (< 90 g/L)	41.38% (12/29)	33.33% (5/15)	χ^2^ = 0.270	0.603
CEA (≥ 10 ng/mL)	10.34% (3/29)	33.33% (5/15)	χ^2^ = 3.512	0.061
Lymphadenectomy (D2)	62.07% (18/29)	100.00% (15/15)	–-	0.008^a^
Lymph nodes harvested	17 (12, 24)	31 (27, 38)	U = 56.0	< 0.001
Positive lymph nodes	2 (1, 5)	3 (2, 8.5)	U = 274.5	0.155
The total blood transfusion volume (mL)	200 (0, 800)	0 (0, 100)	U = 137.5	0.034
The total ICU stay (days)	3 (0, 3)	0 (0, 1.5)	U = 117.0	0.009

^a^ Fisher's exact test

Kaplan-Meier analysis showed that patients in the two-stage group (median OS time is 45 months) had significantly better overall survival than those in the one-stage group (median OS time is 11 months, Fig. 3B, *P*=0.007).

During the perioperative period, patients in the one-stage group received 200 (0, 800) ml of RBC and plasma transfusions. Meanwhile, patients in the two-stage group had only 0 (0, 100) ml (Table 2, Fig. 3C, *P*=0.034). In the one-stage group, patients remained in the ICU for 3 (0, 3) days after surgery. However, patients who underwent two-stage gastrectomy stayed in the ICU for only 0 (0, 1.5) days in total. The results above showed that patients who underwent two-stage gastrectomy had a significantly shorter ICU stay (Table 2, Fig. 3D, *P*=0.009).

### Baseline clinical and pathological parameters before and after PSM

A total of 44 PGC patients who underwent R0 gastrectomy were enrolled, and finally 415 regular GC patients (January 2010 to June 2021) were randomly included in the analysis as a control group. Baseline clinical and pathological parameters are shown in Table 3. Compared with PGC who underwent R0 gastrectomy, regular GC patients had significantly smaller tumor diameter, better tumor differentiation, better T and N stage, and less lower 1/3 of the stomach, lower rate of neoadjuvant chemotherapy and adjuvant chemotherapy (all *P* < 0.05, Table 3).

**Table 3:** Baseline characteristics before and after propensity score matching

Clinical parameters	**Before PSM**			**After PSM**		
	PGC (*n* = 44)	GC (*n* = 415)	*P* value	PGC (*n* = 44)	GC (*n* = 44)	*P* value
Age (years ≥ 70)	29.5% (13/44)	28.2% (117/415)	0.850	29.5% (13/44)	27.3% (12/44)	0.813
Gender ( Male)	75.0% (33/44)	70.4% (292/415)	0.520	75.0% (33/44)	70.5% (31/44)	0.632
Tumor diameter (≥ 5 cm)	72.7% (32/44)	41.2% (171/415)	< 0.001	72.7% (32/44)	82.8% (36/44)	0.309
Tumor differentiation ( SRCC or Poorly)	88.6% (39/44)	68.4% (284/415)	0.005	88.6%(39/44)	86.4% (38/44)	0.747
T staging (T4)	97.7% (43/44)	32.1% (133/415)	< 0.001	97.7%(43/44)	97.7% (43/44)	> 0.999
N staging (N positive)	84.1% (37/44)	58.8% (244/415)	0.001	84.1%(37/44)	86.4% (38/44)	0.764
M staging (M positive)	0.0% (0/44)	1.7% (7/415)	0.385	0.0% (0/44)	0.0% (0/44)	> 0.999
Location (Lower 1/3)	81.8% (36/44)	61.7% (256/415)	0.008	81.8% (36/44)	75.0% (33/44)	0.437
CEA (≥ 10 ng/mL)	18.2% (8/44)	8.7% (40/415)	0.078	18.2% (8/44)	22.7% (10/44)	0.921
Neoadjuvant chemotherapy (YES)	13.6% (6/44)	4.6% (19/415)	0.012	13.6% (6/44)	18.2% (8/44)	0.560
Adjuvant chemotherapy (YES)	100.0% (44/44)	63.6% (264/415)	< 0.001^a^	100.0% (44/44)	100% (44/44)	> 0.999^a^

^a^Fisher's exact test

After PSM, 88 patients (PGC = 44; regular GC = 44) were matched, and the clinical and pathological parameters are shown in Table 3. The result showed that no significant differences were observed between PGC and regular GC groups in terms of age, gender, tumor diameter, tumor differentiation, TNM staging, anatomical location and CEA level and neoadjuvant chemotherapy and adjuvant chemotherapy (all *P*>0.05, Table 3).

### Comparison between propensity score-matched regular GC patients and PGC patients underwent R0 gastrectomy

**Fig. 4:**
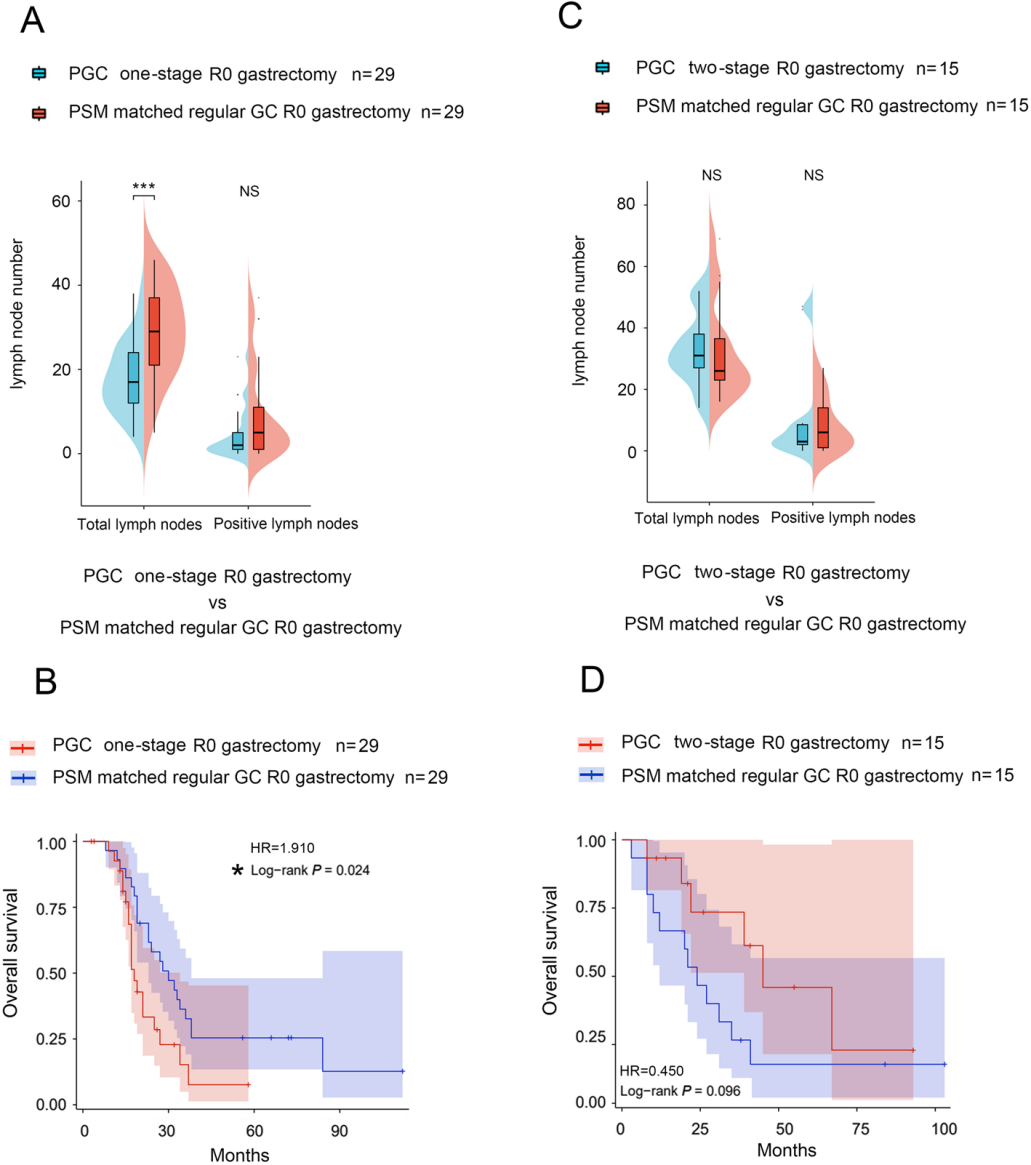
The PGC patients who underwent one-stage R0 gastrectomy had poorer quality lymphadenectomy and worse overall survival compared with propensity score-matched regular GC patients. **A** Compared with propensity score-matched 29 regular GC patients, 29 PGC patients who underwent one-stage R0 gastrectomy harvested significantly fewer total lymph nodes (*P* < 0.001) and fewer positive lymph nodes, but not significantly (*P* = 0.054). **B** Compared with 29 PGC patients who underwent one-stage R0 gastrectomy, 29 propensity score-matched patients with regular GC had a significantly better OS (*P* = 0.024). **C** Compared to 15 propensity score-matched regular GC patients, 15 PGC patients who underwent two-stage R0 gastrectomy had a similar number of total and positive lymph nodes harvested (*P* = 0.506, *P* = 0.787). **D** Compared to 15 propensity score-matched regular GC patients, 15 PGC who underwent two-stage R0 gastrectomy had a similar OS (*P* = 0.096)

**Fig. 5:**
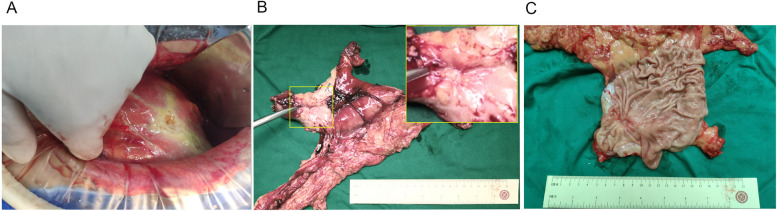
The images of the gross appearance of the perforated gastric cancer and the site of perforation. **A** The surgical image was taken from a patient with perforated gastric cancer. This patient underwent omental patch repair. **B** This patient underwent 3 cycles of SOX chemotherapy. The image of the spacemen of distal gastric shows that the scar on the gastric antrum is the site of the original perforation. **C** Gross appearance of gastric cancer after omental patch repair and 3 cycles of SOX chemotherapy

Subgroup analysis showed that 29 PGC patients who underwent one-stage surgery had significantly fewer total lymph nodes harvested (17 (12, 24) vs 29 (21, 37), *P*<0.001, Fig. 4A) and slightly fewer positive lymph nodes compared with propensity score-matched regular GC patients (*P*=0.054, Fig. 4A). Further analysis showed that 29 PGC patients who underwent one-stage gastrectomy (median OS 18 months) had worse OS than propensity score-matched 29 normal GC patients (median OS 30 months, *P*=0.024, Fig. 4B). Conversely, fifteen-five PGC patients underwent two-stage R0 gastrectomy, harvested a similar number of total and positive lymph nodes (*P*=0.506, *P*=0.787, Fig. 4C), and had a comparable OS to the propensity score-matched regular GC patients (*P*=0.096, Figs. 4D and 5).

## Discussion

Previous studies show that the most common cause of gastric perforation is benign peptic ulcer, and gastric perforation caused by gastric cancer is relatively rare. Gastric cancer accounts for only about 10–16% of patients with gastric perforation [3, 4].

If a gastrointestinal perforation is diagnosed, emergency surgery must be performed immediately. Due to the urgency of treating peritonitis, some of the necessary preoperative imaging or pathological tests are often omitted. Therefore, PGC is rarely diagnosed preoperatively. Even if it is diagnosed preoperatively or intraoperatively, the local infiltration and lymph node metastasis of gastric cancer are often misjudged due to the influence of abdominal infection [3]. Therefore, it is still difficult to accurately assess the stage of gastric cancer and design the best surgical strategy. Furthermore, as most of these patients have a severe abdominal infection, even with unstable vital signs, standard D2 radical assisted gastrectomy can rarely be performed in an emergency situation.

In this study, only seven patients had pathological results before the onset of perforation. Of all PGC patients who achieved R0 resection, only 62.07% achieved D2 resection during the initial emergency surgery. However, in the two-stage gastrectomy group, where radical gastrectomy is performed after initial treatment of peritonitis, all patients achieved D2 resection.

In western countries, D2 lymphadenectomy was not considered a standard procedure. In eastern countries, however, D2 lymphadenectomy has been adopted as standard clinical practice [8, 9]. Recent randomised controlled trials have shown that D2 radical surgery for gastric cancer can improve disease-specific survival in patients with advanced disease [10, 11]. The academic consensus is that D2 radical resection for PGC patients improves treatment outcome. However, in an emergency situation, it is difficult to perform D2 radical surgery due to poor general condition such as perioperative sepsis and underlying basic diseases. Besides, such radical surgery can potentially cause damage to the lives of PGC patients in poor general condition. Thus, some researchers advocate two-stage D2 radical gastrectomy, i.e., one-stage perforation repair for perforation, followed by radical surgery after the patient's overall condition has improved and accurate pathological staging has been performed. While awaiting radical gastrectomy, chemotherapies can be given according to the patient's molecular pathology results.

Several reports on patients with PGC have shown that those who undergo curative resection have a significantly better prognosis than those who undergo non-curative resection [12]. Lehnert et al. [3] suggested that oncological surgery for gastric cancer should be considered once the patient has recovered from peritonitis. Two-stage surgery is a better option, especially for frail people with resectable PGC [13]. Hata et al. [14] demonstrated that patients who underwent emergency one-stage gastrectomy had significantly lower rates of R0 resection and D2 lymph node dissection than those who underwent two-stage gastrectomy. In addition, patients who underwent two-stage gastrectomy had a significantly lower mortality rate than those who underwent one-stage gastrectomy. Although Hata's study included a relatively large number of GC patients, it has some shortcomings. As a retrospective study, all the cases in the study came from case reports obtained from the Japan Medical Abstract Society database. All the cases came from different medical centres in Japan and other countries and spanned more than 30 years. Therefore, the consistency of surgery, pathology, and follow-up of patients in this study was not satisfactory. In addition, it is difficult to continue follow-up if a patient has tumor recurrence or tumor-related death after the case reports are published. Therefore, the survival curve in this article is partially flawed. At the same time, this study did not look at the number of lymph nodes removed during surgery and did not compare the number of lymph nodes removed in PGC and regular GC surgery.

According to the findings of this study, the OS of patients with PGC who can achieve R0 resection after two-stage surgery is better than those who underwent one-stage radical gastrectomy. In addition, two-stage surgery can obtain significantly more lymph nodes, fewer perioperative blood transfusion and shorter ICU stay. These results show that two-stage surgery has a lower likelihood of postoperative complications, a shorter recovery time after radical gastrectomy, and a better long-term prognosis. The PSM analysis confirmed the above findings from a different perspective. Compared with the propensity score-matched regular GC patients, PGC patients who underwent one-stage gastrectomy had a worse quality of lymphadenectomy and suffered a worse OS. Conversely, two-stage gastrectomy achieved a comparable quality of lymphadenectomy and a similar OS compared to propensity score-matched regular GC patients.

Clinical trials have shown that perioperative chemotherapy has potential benefits for patients with locally advanced gastric cancer, including the MAGIC, ACTS-GC and CLASSIC trials [15–17]. Recently, the RESONANCE trial led by Chinese investigators demonstrated that the S-1 plus oxaliplatin (SOX) regimen as neoadjuvant chemotherapy was associated with an increased rate of D2 lymph node dissection and R0 resection, suggesting that the SOX regimen used prior to D2 gastrectomy may prolong median survival, DFS and OS [18]. In this study, six patients received neoadjuvant chemotherapy prior to curative surgery, and 3 of them achieved a more than 20 months disease-free survival; one patient survived over 90 months. These preliminary findings suggest that neoadjuvant chemotherapy probably play an important role in prolonging OS. However, due to the small number of patient samples observed, the current conclusions are not conclusive. A firm conclusion awaits further evidence from studies with larger sample sizes.

R0 resection refers to a microscopically margin-negative resection in which no gross or microscopic tumor remains in the primary tumor bed, and this definition was provided by Hermanek 28 years ago [19]. If this definition is correct, R0 resection should be associated with high survival and low recurrence. In reality, however, R0 resection has not shown such a desirable prognosis. The Hermanek definition does not follow this scenario because it is mainly concerned with the primary tumor site and not with the details of lymphatic spread. It is therefore reasonable to assume that less extensive lymphadenectomy will result in understaging of GC patients. In addition, less extensive lymphadenectomies are more likely to miss metastatic lymph nodes [20]. Conversely, extensive lymphadenectomy in patients with stage N3 gastric cancer may result in less loco-regional recurrence and better survival outcomes [21].

The retrospective analysis of 40,281 GC patients from the American National Cancer Database showed that the number of harvested lymph nodes ≥ 29 in the patients with higher stages GC provides a comparable OS to those with lower stage GC [22]. Therefore, the 8th AJCC recommends the examination of at least 16 retrieved lymph nodes for better staging, but if possible, ≥ 30 lymph nodes are preferred for accurate staging and prognosis [23, 24]. We need to be aware that lymph nodes are collected in different ways by different medical institutions, surgeons and pathologists [25]. Therefore, to increase the number of lymph nodes harvested, surgeons should separate lymph nodes ex vivo from the gastrectomy specimen before sending them to pathologists, and require pathologists to evaluate as many lymph nodes as possible, with a target of 25 or more [26]. Thus, good pathological diagnosis can more accurately assess the pathological stage of patients. In our study, we found that the number of lymph nodes dissected was closely related to patient prognosis. Our results showed that two-stage surgery provides a better quality of lymph node dissection, and provided a better OS than one-stage surgery. Additionally, compared with regular GC patients after PSM, the OS of PGC patients underwent two-stage surgery was similar to that of regular GC patients. Therefore, we speculate that a good quality of lymph node dissection may benefit the overall survival of patients.

Recurrence of the tumor is associated with survival of patients with GC. The types of recurrence after radical resection of gastric cancer include local recurrence, peritoneal implantation, lymph node metastasis, and haematogenous metastasis. The pattern of recurrence in patients with PGC remains controversial. A previous study showed that the rate of peritoneal metastases after curative resection was significantly higher in T4 patients [27]. However, in a system review showed that there were no significant differences in the rate and pattern of recurrence between perforated and non-perforated gastric cancer when curative surgery was performed [28]. This study is a real-world retrospective study, in which patients came from 13 different medical centers, and the time span was 12 years. Considering the inconsistent quality of CT reports and other unavoidable non-objective factors, we did not focus on patients' recurrence patterns to ensure the scientific validity of our study.

In recent years, NCCN guidelines have recommended that diagnostic laparoscopy and cytology should be considered in GC patients with T1b disease or higher (category 2B). In the version 2.2022, NCCN gasrric cancer guidelines, for locoregional (cM0) patients, this recommendation was changed as follows: Medically fit, surgically unresectable: “consider laparoscopy with cytology” changed from category 2B to category 2A [29]. For the patients who without visible peritoneal metastases, cytological examination of abdominal lavage fluid can reveal the presence of free intraperitoneal cancer cells, and hyperthermic intraperitoneal chemotherapy (HIPEC) therapy could significantly reduce postoperative tumor recurrence and improve survival [5, 30]. Because the concept of cytology was not widely accepted in the early years, cytology was performed in only 4 PGC patients who underwent two-stage gastrectomy, and none of them had a positive peritoneal lavage cytology (CY1). And only two patients who underwent R0 surgery received intraoperative extended-release fluorouracil implants after radical resection.

Overall, this study provides preliminary answers to some of the questions about treatment options for PGC. For PGC patients in poor condition, two-stage treatment is a better option if D2 radical gastrectomy cannot be achieved in emergency surgery. This study has several limitations. Firstly, the sample size of this retrospective study was limited due to the rarity of PGC. In addition, the PGC patients in this study came from many different medical institutions, and the methods of collecting lymph node samples from the specimens were not uniformly standardised. Because of these limitations, prospective clinical trials are needed in the future.

## Conclusions

For PGC patients in poor condition, two-stage treatment is a better option when D2 radical gastrectomy cannot be achieved in emergency surgery. Therefore, two-stage gastrectomy may be a promising procedure for PGC patients.

## Data Availability

The data that support the findings of this study are available on request from the corresponding author.
